# Preoperative evaluation of transcatheter aortic valve replacement with assistance of 3D printing technique: Reanalysis of 4 death cases

**DOI:** 10.1016/j.jimed.2019.10.006

**Published:** 2019-10-23

**Authors:** Hao Zhang, Yu Shen, Lei Zhang, Chao Song, Zaiping Jing, Qingsheng Lu

**Affiliations:** a Department of Vascular Surgery, Changhai Hospital, Navy Military Medical University, 168Changhai Road, Shanghai, 200433, PR China; b Department of General Surgery, No. 202, Hospital of People’s Liberation Army, 5Guangrong Road, Shenyang, Liaoning, 110812, PR China

**Keywords:** TAVR, 3D printing, HeartPrint® flex, Aortic stenosis, In vitro test

## Abstract

**Introduction:**

Transcatheter aortic valve replacement (TAVR) can have some fatal complications during and after the operation. Until recently, pre-procedural imaging with cardiac computed tomography (CT), which is required to evaluate for TAVR, had its own imperfections. We aimed to determine whether 3D printed models can predict complications when other pre-procedural imaging techniques failed.

**Methods:**

Vascular center patients with aortic valve stenosis, who died after TAVR between June 2011 and June 2016, were enrolled in this retrospective study. The CT datasets of the patients were imported into a three-dimensional (3D) construction software and then printed by flexible material. To predict complications during and after operations, we designed a release test using the non-valved stent mode that was consistent with the Edwards Sapien XT valve in size and radial support force.

**Result:**

The 3D model predicted the coronary obstruction and annular rupture in the in vitro release process, which was consistent with what happened in the actual operation.

**Conclusion:**

Three-dimensional modeling facilitates pre-operative assessment of patients receiving TAVR, with accurate simulation of intraoperative status.

## 1 Introduction

Transcatheter aortic valve replacement (TAVR) has already been used as a standard treatment for patients with symptomatic severe aortic stenosis (AS). Pre-procedural cardiac computed tomography angiography (CTA) is required to simulate the annulus sizing.^1^ However, several limitations of CTA have been reported: 1) The valve annulus structure can only be measured through a certain plane, and the three-dimensional (3D) structure can not be completely reflected; 2) It cannot assess the status after implantation, such as relative movement of the annular calcification and changes of the coronary ostium direction and height; and 3) It cannot predict intraoperative and postoperative complications accurately.^2^^,^^3^ Recently, 3D printing has been used for establishing individualized anatomy models for pre-operative planning.^4^ This study aimed to determine whether 3D printed models could be used for predicting complications when other pre-procedural imaging techniques failed.^5^

## 2 Methods

### 2.1 Study population

Our vascular center patients with aortic valve stenosis, who died after TAVR between June 2011 and June 2016, were enrolled in the study. We retrospectively collected the data of 4 patients in total, whose pre-TAVR cardiac CTA were available and indicated no fatal complications. The preliminary causes of the death proved to be 2 coronary obstructions and 2 annular ruptures (Table 1).

**Table 1:** Patient parameters before the operation by CTA.

Patient	CSA (mm^2^)	Annulus diameter (mm)	Distance between annulus and coronary ostium (mm)		Valve size (mm)	Complication
			left	right		
1	618	28	11.1	16	29	Left coronary obstruction
2	276	18.7	19.9	12	23	Left coronary obstruction
3	491	25	9.47	11.2	26	Annular rupture
4	607	27.8	11.5	14.4	29	Annular rupture

All patients in the study received balloon-expandable Edwards Sapien XT valve treatment. Prosthesis size was selected using the SAPIEN sizing calculator, based on the annulus diameter calculated from the annular cross-sectional area (CSA) measured by cardiac CTA.

### 2.2 Pre-procedural cardiac CTA

All patients’ cardiac CTA were performed under an inspiratory breath-hold. The electrocardiogram (ECG) was recorded simultaneously to allow retrospective gating through the heart over a complete RR interval (0–99%) by every 5% time phase using a 320 × 0.5 mm detector row scanner CT (Toshiba Aquilion ONE Dynamic Volume CT). Patients received about 50 ml iodinated contrast, followed by 40 ml normal saline solution at a rate of 5 ml per second.

### 2.3 Creation of 3D models

In efforts to develop a better preoperative assessment and a more accurate forecast for postoperative complications, the 4 cases of those who died during the TAVR procedure were re-analyzed using 3D models based on CTA data. These 3D models were created by selecting the template at the RR interval of 30%–40% (systolic phase) during the maximal opening of the aortic valve.^6^

The CTA datasets of the patients were imported into a 3D construction software (Mimics, Materialise). The corresponding threshold values for each patient were adjusted to capture the individual contrast range and to semi-automatically exclude the calcium. The blood pool included aortic root, annulus, and left ventricular outflow tract. The calcium was segmented manually. Segmented models were then imported into the computer-aided design software (3-matic, Materialise) to reconstruct the blood vessel wall and aortic valve. A 2-mm thick wall was added to the outside of the blood pool in all models, and a 1-mm aortic valve was added to aortic root; this strategy was necessary because the true aortic wall and valve leaflets were too thin to segment. Finally, the blood vessel wall, valve leaflets, and calcium were combined into one model named the aortic root model. The models were converted to 3D printable standard tessellation language (STL) files.

The aortic models were printed using a 3D printer, blood vessels and valves were printed with flexible material (HeartPrint® Flex), and calcium was printed with a rigid white material. As both Young’s modulus and distensibility of the HeartPrint® Flex model are in compliance with human arterial tissue properties, the designed model not only offers the correct geometry of the aorta, but also mimics the material behavior of the real anatomy.

### 2.4 Release test in 3D models

In order to predict complications during and after operations, we designed a release test using the non-valved stent mode that was consistent with the Edwards Sapien XT valve in size and radial support force. A 0.035-inch guidewire from the ascending aorta was put into the left ventricular outflow tract through the aortic valve. The pre-dilation balloon in the corresponding size was selected and placed at the annulus level, and valvuloplasty was performed. The stent model was delivered to the annulus level and implanted by injecting the balloon with the same amount of normal saline that is used in the actual operation.

The whole process was observed and recorded by endoscope, especially the valve leaflets and annulus, so that we can know the anatomic relationships between the prosthetic valve and the aortic valve complex, such as coronary ostia, calcification, etc. after the operation. The presence of coronary occlusion was confirmed when the edge of the prosthetic was higher than the coronary ostia or when the calcification was pushed towards the coronary ostia. As the HeartPrint® Flex printed model biomimetically imitated the aorta, the presence of aortic annular rupture was defined as the rupture of the model during or after the process.

## 3 Results

### 3.1 Patient 1

#### 3.1.1 Pre-operation

The calcification was obvious in the left ventricular outflow tract side of the valve. The calcification ridge was observed in the junction of non-coronary and right coronary valves, and the calcification was visibly attached to the edge of the left coronary valve leaflet.

#### 3.1.2 Intro-operation

**Fig. 1:**
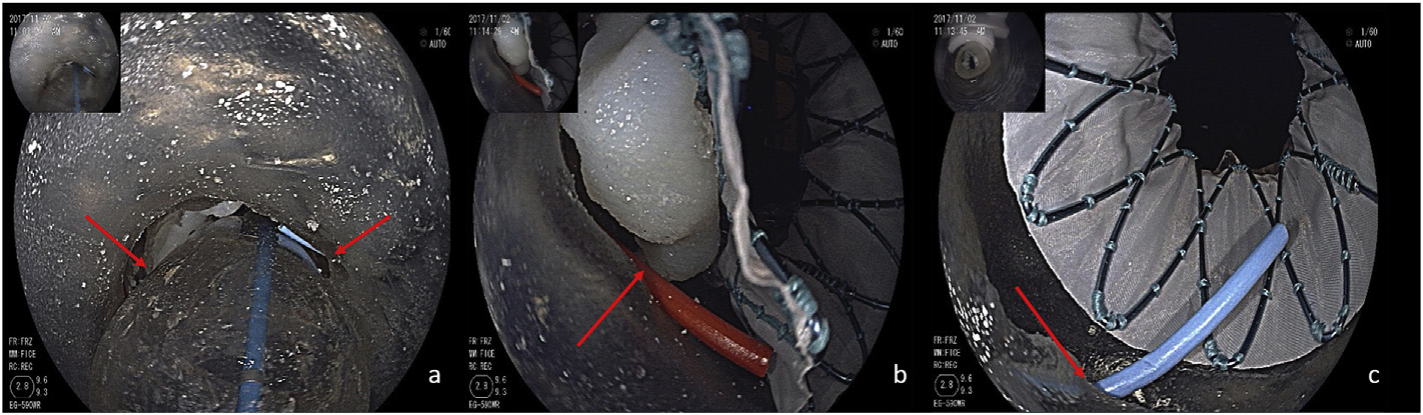
After the balloon valvuloplasty, the left and right coronary ostia(red arrows) were intact(a), after the THV was implanted at the annulus plane, the calcification clump of the leaflet obviously blocked the left coronary ostia (b), while the right ostia was intact (c). (For interpretation of the references to colour in this figure legend, the reader is referred to the Web version of this article).

After the balloon valvuloplasty, the left and right coronary ostia (red arrows) were visibly intact (Fig. 1a).

#### 3.1.3 Post-operation

After implanting the stent, it was clearly observed from the ascending aorta side that the left coronary valve was pressed towards the annulus after stent expansion; the calcification clump of the leaflet obviously blocked the left coronary ostium (Fig. 1b), while the right ostium was not affected (Fig. 1c).

### 3.2 Patient 2

#### 3.2.1 Pre-operation

The calcification was evidently on the side of the valve leaflet which faced towards the ascending aorta, especially in the right coronary valve. Calcification was not observed in the left ventricular outflow tract.

#### 3.2.2 Intro-operation

The valve structure remained intact after the balloon valvuloplasty.

#### 3.2.3 Post-operation

**Fig. 2:**
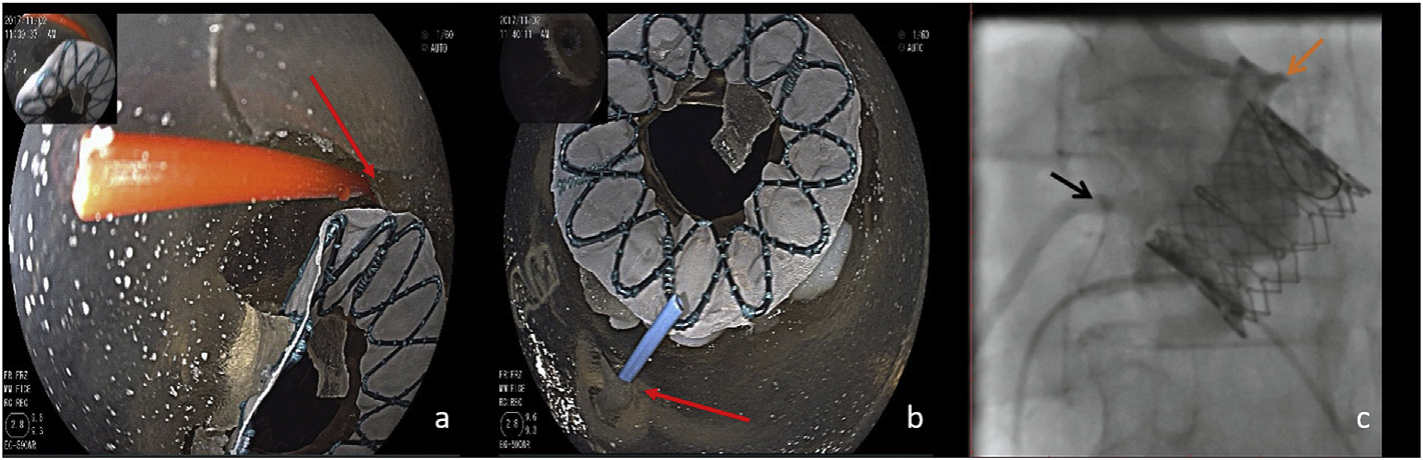
The left coronary ostia was partially covered by the distal edge of prosthesis (a), the right coronary ostium was not influenced (b), compares favourably with the DSA during the operation (c).

The stent was accurately positioned. The distal edge of the stent could be harmful to the left coronary ostium, which was partially covered after the implantation (Fig. 2a). The right coronary ostium was not influenced by the stent structure (Fig. 2b).

### 3.3 Patient 3

#### 3.3.1 Pre-operation

The valve was in good condition as observed from the ascending aorta side. The patient could be defined as bicuspid Type 0 from the left ventricular outflow tract. The calcification plaques could be seen at the left coronary leaflet.

#### 3.3.2 Intro-operation

**Fig. 3:**
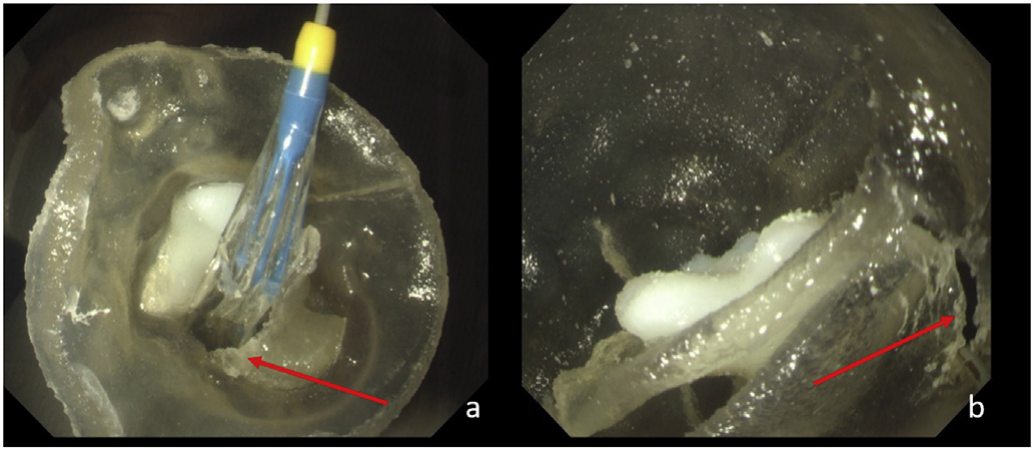
After the balloon valvuloplasty, the annulus was rupture at a slight degree (red arrows). (For interpretation of the references to colour in this figure legend, the reader is referred to the Web version of this article).

The annulus displayed rupture to a certain degree after the pre-expanding balloon inflated to the largest extent, while the overall shape of the valve remained after the balloon withdrawal (Fig. 3).

#### 3.3.3 Post-operation

The stent was well embedded, and the valve avulsion was clear while the shape was changed. Stent displacement occurred due to the annulus rupture, and both left and right coronary arterial openings were partially covered. Five minutes after releasing, the annulus model was torn from the annulus to the ascending aorta.

### 3.4 Patient 4

#### 3.4.1 Pre-operation

**Fig. 4:**
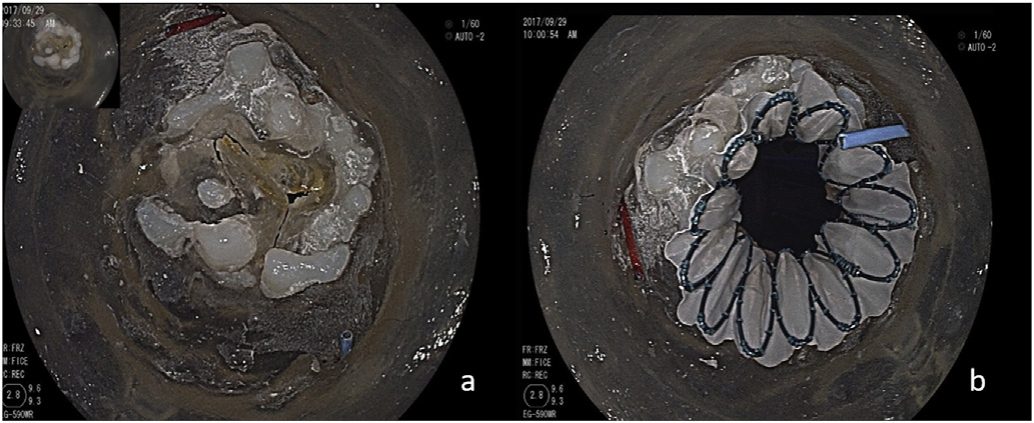
Calcification was apparent in the surrounding area of the annulus and the junction of the left (blue wire) and right (red wire) coronary valve(a); The THV was decentration after implanted, cause of the calcification. The left (blue wire) and right (red wire) coronary ostia were intact. (For interpretation of the references to colour in this figure legend, the reader is referred to the Web version of this article).

The patient was clearly defined as bicuspid Type 1. Calcification was also apparent in the surrounding area of the valve annulus and the junction of the left and right coronary valves, where the calcification ridge was formed (Fig. 4a).

#### 3.4.2 Intro-operation

The annulus did not show obvious rupture after the pre-expanding balloon inflated to the largest extent, and the overall shape of the valve remained after the balloon withdrawal.

A 29 mm non-valved stent was successfully transferred to the annulus by the guiding wire through the delivery system. After injecting 33-cc water by the pressure pump, the balloon expand successfully. The junction of the right and left coronary valves showed evidence of a tear, and a partial tear also appeared between the non-coronary and right coronary valves. The stent was released at the annular plane (Fig. 4b).

#### 3.4.3 Post-operation

The stent was well placed. The red guiding wire barely entered the aorta through the left coronary artery, which was partially covered by the stent and the valve. While the blue guiding wire entered the aorta root via the right coronary artery, which was also partially covered, it could barely pass through the gap between the stent and the coronary sinus. The original partial tear at the junction of the right and non-coronary valves led to an annulus rupture that pointed from the annulus to the ascending aorta due to the persistent existence of the stent.

## 4 Discussion

### 4.1 The utilization of a 3D model in pre-operation evaluation

CTA assesses the annulus structure in a plane that is formed by the aortic cusp hinge points of the three valve leaflets.^7^ This method can only realize the planar evaluation instead of a 3D evaluation, thus it cannot accurately assess the pathological structure of the annulus and leaflets.^8^ For instance, patient three was Type 0 bicuspid with only 2 valve leaflets, which cannot form a plane with their aortic cusp hinge points, thus the planar measurement by CTA could not be realized.^9^ Supra-valvular aortic stenosis, valve hypertrophy, and sub-valvular obstruction can cause the narrowest plane to be off the annulus after the stent release. Therefore, the actual conditions of the narrowest plane in the surrounding area of the aortic valve cannot be fully assessed by CTA.

A 3D model can precisely mimic related structures from the aortic root to the left ventricular outflow tract.^10^ In addition to depending on the diameter, circumference, and area, with consideration of various factors and in vitro releasing results, the valve annulus structure can be measured under direct observation. By integrating the valve condition after releasing, a 3D model works on the selected anchoring plane and simulates the potential complications caused by the discrepancy in shape (the diameters are the same) in and after the operation.

The location and direction of the coronary ostia would change after placing the prosthesis into the annulus due to the structure itself and the calcification; therefore, a CTA is unable to predict the intra-operation and post-operation situations. A 3D model can reflect the complex structures of the annulus and leaflets. In combination with in vitro release, it can simulate the operation, imitate the relative movement of the coronary ostia and calcification, and may predict the intra- and post-operative risks. The advantage of a 3D model is its ability to expose the potential risks of the coronary artery obstruction.

CTA evaluates the risks of the coronary artery by comparing the prosthesis height with the vertical distance from the coronary ostia to the annulus plane. Although the annulus will expand in a parallel manner, the study showed that the possibility of annulus expansion was very small.^2^ The relative distance between the coronary ostia and the annulus plane as well as the angle between coronary flow direction and the aorta would change after implantation, which further influences the coronary blood supply.

A 3D printed model can print the coronary artery to observe the artery flow direction and shape as well as analyze the relationship between the coronary artery and the aorta root. Incorporating the in vitro experiment results, a 3D model can predict the change of the coronary artery after implantation.

### 4.2 Calcification-related risk analysis by 3D model

It is evident that the calcification around the annulus, especially the calcification with sharp morphology, wide range and high ridge, is an important factor in the cause of annulus rupture, coronary artery obstruction, displacement of prosthesis, and other severe complications.^11^^,^^12^ The risk of calcification after implantation cannot be evaluated by traditional imaging. Presently, CTA can only realize the initial assessment via parameters like calcification score, size, ridge height, and location.

**Fig. 5:**
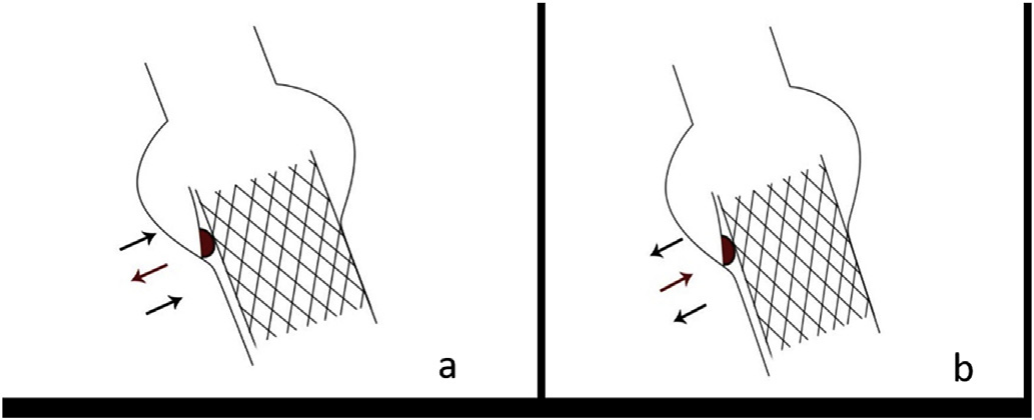
The calcification was pushed outward (a) and inward (b) relative to the annulus.

The application of a 3D model in the in vitro release is feasible and beneficial in observing the relative motion of calcification during and after the operation. Based on the observations in this study, 2 kinds of calcification movements were presented:

1) Affected by the stent radial supporting force, the calcification combined loosely with the surrounding tissues and moved outward with the stent expansion. Due to the firm connection between the calcification and surrounding tissue, the calcification remained relatively static during the stent expansion process. 2) At the same time, the calcification blocked the stent expansion and led to the outward movement of the annulus tissue on other sides, which was pushed by the expanding stent. As a result, the calcification moved relatively inward (Fig. 5).

The calcification clump on the leaflet may be pushed towards the coronary sinus and cause obstruction of the coronary ostium. The correlation between the calcification motion and annulus rupture requires further mechanical experimental analysis and in vitro experimentation to demonstrate. However, it is undoubted that a 3D model can better restore the relative motion of the calcification.

## 5 Application of a 3D model

As reported by previous literature and in this study, a 3D model has significant advantages over CTA in terms of the assessment of important factors such as abnormal calcification, annulus plane, and coronary artery risks; it can predict the risks during and after the operation.^13^ A 3D model is applicable to surgeries with higher risks, especially for patients with complex annulus and leaflet structures, severe calcification, and poor coronary artery conditions.

Incorporating the in vitro experiment, we may avoid risks by reducing the prosthesis size, changing the surgical path, or the type of prosthesis. These trial methods can also be verified in in vitro experiments.

## Limitation

We acknowledge that our results present a partial view into the potential advantages of a 3D printed model in the pre-operative assessment of TAVR. The utilization of a 3D printed model in TAVR requires further mechanical experimental analysis with more patients.

## 6 Conclusion

Many TAVR patients have structural heart disease, requiring CTA for assessment; however, CTA cannot suggest some potential procedural risks. The 3D model facilitates pre-operative assessment of patients receiving transcatheter aortic valve replacement, with accurate simulation of intraoperative status and analysis of the operation risks.
